# Psychometric Properties of the Multidimensional Health Locus of Control Scale Form C in a Non-Western Culture

**DOI:** 10.1371/journal.pone.0107108

**Published:** 2014-09-09

**Authors:** Barna Konkolÿ Thege, Beatrix Rafael, Magda Rohánszky

**Affiliations:** 1 Department of Psychology, University of Calgary, Calgary, Canada; 2 Firebird Foundation, Budapest, Hungary; 3 Institute of Behavioral Sciences, Semmelweis University, Budapest, Hungary; 4 Psychiatric Clinic, University of Szeged, Szeged, Hungary; 5 Special Hospital of Chest Illnesses, Deszk, Hungary; 6 Department of Oncology, Szent László Hospital, Budapest, Hungary; Georgia State University, United States of America

## Abstract

Form C of the Multidimensional Health Locus of Control Scales (MHLC-C) was designed to investigate health-related control beliefs of persons with an existing medical condition. The aim of the present study was to examine the psychometric properties of this instrument in a culture characterized by external control beliefs and learned helplessness—contrary to the societal context of original test development. Altogether, 374 Hungarian patients with cancer, irritable bowel syndrome, diabetes, and cardiovascular and musculoskeletal disorders were enrolled in the study. Besides the MHLC-C, instruments measuring general control beliefs, anxiety, depression, self-efficacy, and health behaviors were also administered to evaluate the validity of the scale. Both exploratory and confirmatory factor analytic techniques were used to investigate the factor structure of the scale. Our results showed that the Hungarian adaptation of the instrument had a slightly different structure than the one originally hypothesized: in the present sample, a three-factor structure emerged where the items of the Doctors and the Others subscales loaded onto a single common component. Internal reliability of all three subscales was adequate (alphas between .71 and .79). Data concerning the instrument's validity were comparable with previous results from Western countries. These findings may suggest that health locus of control can be construed very similarly to Western countries even in a post-communist society—regardless of the potential differences in general control beliefs.

## Introduction

Health-related locus of control refers to an individual's beliefs or expectations regarding which persons or other factors determine his or her health [1]. Throughout the past decades, a large body of literature has been devoted to the investigation of the role of health-related control beliefs in determining a number of aspects of health and illness. For instance, previous findings revealed that perceived health-related control influences the course of chronic diseases [2] and health behaviors in both healthy [3] and ill populations [4]. Health-related control beliefs were also linked to adherence with treatment regimens [5], [6] and adjustment to chronic diseases [7] and could explain in part the variance regarding ethnic differences in mental disorders (e.g., depression) [8].

To measure health-related control beliefs, the vast majority of studies has used different forms of the Multidimensional Health Locus of Control (MHLC) Scales developed by Wallston and colleagues [9]. While the A and B forms were constructed to measure general health-related control beliefs without being specific to any health behavior or condition, Form C (MHLC-C) was developed to investigate health-related control beliefs of individuals with an existing medical condition [1]. Contrary to the A and B forms of the MHLC Scales, relatively few studies have investigated the psychometric properties of Form C; this is particularly true for non-English speaking countries. Of the few investigations conducted in these countries, Italian [10] and Swedish [11] examinations confirmed the hypothesized four-factor structure, reliability, and validity of the instrument, while findings from China [12], [13] consistently disaffirmed the psychometric adequacy of the instrument.

A potential explanation for these discrepancies could be explained by the prominent cultural differences between these regions of the world: while social arrangements of the Western European countries—where the psychometric properties of the instrument proved to be adequate—are pretty close to those of the original test development, in China, the cultural context is substantially different. Therefore, to clarify whether Form C of the MHLC Scales should be considered an instrument adequate only for Western societies or whether it can be used with confidence to investigate health-related control beliefs in non-Western countries as well, it seems plausible to investigate the psychometric properties of this instrument in more societies without strong democratic traditions.

Similarly to China, individuals in Eastern European countries lived under totalitarian political regimes for decades in the second half of the twentieth century, which dramatically increased the occurrence of hopelessness and learned helplessness in these societies [14]–[17]. Compared to North America and Western European democratic states, people in communist countries—including China, Eastern Europe and the former Soviet Union—had less control over their personal lifestyles and lives in general, and were characterized mainly by external control beliefs [18]–[20]. Although the dictatorial political system was abolished in Eastern Europe around 1989 bringing with it basic political and economic changes, the helpless attitude and strong external control beliefs have not yet disappeared [21]–[23].

Does this social climate also affect health-specific control beliefs? Does the factor structure of the MHLC-C in the understudied post-communist European societies differ from that being established in countries with strong democratic traditions where people feel more in control of their lives in general? The aim of the present study was to answer these questions by presenting the development of an Eastern European (Hungarian) adaptation of the MHLC-C and by providing data on its psychometric properties within this unique socio-cultural context. In addition to the adaptation of the scale to the Hungarian culture, our intention was to contribute to the better understanding of the significance of cultural differences concerning health-related control beliefs.

## Materials and Methods

### Sample and procedure

The protocol of the present study was approved by the Ethical Committee of Szent László Hospital, Budapest (15/EB/2010). Participants were recruited by institutional psychologists from four hospitals in different cities in Hungary including the capital (Budapest) and three medium-sized cities (Szeged, Kecskemét, and Pécs) in different regions of the country. Data collection was conducted between June 2010 and July 2012. Participation was voluntary; respondents were informed about the purpose of the study and gave written consent to the anonymous utilization of their data.

Altogether, 374 patients were enrolled in the study (125 cancer patients, 121 patients with cardiovascular disease, and 128 patients with musculoskeletal diseases, diabetes, or irritable bowel syndrome). The mean age of the participants was 53.6 years (SD = 12.5 years) with more women (*n* = 221; 59.4%) than men (*n* = 151; 40.6%) participating in the study. Educational distribution of the sample was relatively balanced: 115 (34.1%) individuals had completed elementary school, 135 (40%) persons completed the middle level of schooling, and 87 (25.8%) persons completed an upper level education.

### Measure instruments

Form C of the Multidimensional Health Locus of Control Scales consists of four subscales: Internal (six items), Chance (six items), Doctors (three items), and Others (three items), assessing the extent to which respondents believe the given factors affect their health status or progress of their disease. Participants rated the degree of their agreement on a 6-point rating scale ranging from 1 (strongly disagree) to 6 (strongly agree). Three independent translators (psychologists with master's or doctoral level education) translated the original English-language version of the scale from English to Hungarian. This step was followed by the development of a consensual version by the same persons. Then, a back translation was prepared by an additional bilingual professional (a psychologist with a master's level education), which was found to be substantially identical to the original version by a fifth independent colleague (with a background of social work). The full text of the Hungarian version of the MHL-C is available in a supporting information file (Text S1) on the publisher's website.

To evaluate the validity of the Hungarian version of the MHLC-C, we examined the correlation of its subscales with distinct but theoretically related constructs. To facilitate international comparisons, we attempted to employ the same or very similar constructs and measure instruments as were used in previous studies investigating the psychometric properties of the scale [1], [11], [24].

General locus of control was assessed by the Hungarian version [25] of Levenson's Locus of Control Scale [26]. Internal consistency of all three, 8-item subscales was adequate in the present sample (Cronbach's alphas of .71, .81, and .83 for the Internal, Chance, and Powerful Others subscales, respectively). Anxiety and depression was assessed by the Hungarian version [27] of the Hospital Anxiety and Depression Scale [28]. Both 7-item subscales of this instrument showed good reliability coefficients (alphas of .83 and .87 for the Anxiety and the Depression subscales, respectively). To assess self-efficacy, the Hungarian version [29] of Schwarzer's Generalized Self-Efficacy Scale was employed [30]. This 4-item instrument also had good internal consistency (Cronbach's alpha  = .84).

To estimate participants' subjective evaluations of their health status, two questions were used. The first referred to illness intrusiveness: “Taken as a whole, to what degree does your disease affect your everyday life?” Participants provided answers on an 8-point rating scale ranging from 0 (not at all) to 7 (completely). The second question assessed general self-rated health: “How would you estimate your current state of health?” (1 =  very bad, 2 =  bad, 3 =  average, 4 =  good, and 5 =  excellent).

Health behaviors were evaluated by six questions. Nutritional habits were assessed by the question “Generally, to what extent do you pay attention to eating healthily?” Dental hygiene was evaluated by asking “To what extent do you pay attention to the health of your teeth?” In both cases, a 5-point rating scale was used ranging from “not at all” to “completely.” Smoking status was measured by the item “Do you consider yourself as a non-smoker/an occasional smoker/a daily smoker?' Drinking habits were evaluated by asking “In the past twelve months, how often did you drink five or more drinks per occasion (1 drink  = 3 dl of beer or 2 dl of wine or 0.5 dl of spirits)?” The five response options were as follows: never/once or twice/three to six times/seven to 10 times/more than 10 times. Physical activity was assessed by the item “How often do you do any sort of exercise like swimming, running, cycling or playing football?” with four response options: never, rarely, weekly, and several times a week. Finally, proneness to seek medical attention was evaluated by asking “When you have any health concerns, do you turn to a health professional immediately?” Again, five options were offered: never, rarely, often, most often, and always.

### Statistical analyses

AMOS 21 and SPSS 21 software was used to conduct the statistical analyses. Data of those respondents who had more than six missing values for the 18-item MHLC-C were excluded from the analyses (n = 8; 2.14% of the total sample). Remaining missing values for the MHLC-C were filled by regression imputation using maximum likelihood estimation by AMOS [31] before conducting the factor analysis (in the case of 45 respondents, 12.0% of the sample). Since the distribution of the continuous variables proved to be non-normal according to the Shapiro-Wilk W statistics, methods robust for the violation of multivariate normality were employed.

In order to evaluate the factor structure of the instrument, both exploratory and confirmatory factor analytic techniques were used. When conducting the confirmatory factor analysis, the maximum likelihood estimation was used and the Bollen-Stine bootstrap modification was employed to adjust for the violation of normality. Since subscales of the MHLC Form C were reported to correlate with each other [1] and because this method does not require the extracted factors to be independent, the oblimin rotation was chosen when conducting the principal component analysis. To determine the number of components to retain, a parallel analysis was conducted [32]. This technique is a Monte Carlo-based simulation method that compares the eigenvalues from the study sample with those obtained from a random data set. A component is to be retained if its eigenvalue exceeds the one derived from the 95th percentile of the random sample. Random eigenvalues for the comparisons were generated using a web based application [33].

Internal reliability of the MHLC-C was evaluated by calculating Cronbach's alpha coefficients. Interrelationships among the continuous and ordinal variables were analyzed by calculating Spearman correlation coefficients. Differences in the strength of these relationships were evaluated by a web-based application calculating Steiger's *Z* scores [34]. Since the third patient group (individuals suffering from musculoskeletal diseases, diabetes, or irritable bowel syndrome) was too heterogeneous to draw meaningful inferences from their inclusion in the group comparisons, only the two more homogenous patient groups (cancer and cardiovascular patients) were compared along the MHLC-C subscales using the Mann-Whitney U-test. In this case, effect size *r* was calculated using the following formula: 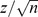.

Following the recommendations of the original test authors and others [24], [35]–[37], when investigating the relationships of the MHLC-C domains with health behaviors, an aggregated index was calculated and used instead of examining single health behaviors (with the exception of the item measuring the proneness to seek medical attention, which was employed independently to assess the validity of the Doctors subscale of the MHLC-C). The index was created following the procedure described by Paine and colleagues [36]. Answers to questions referring to healthy diet, dental hygiene, smoking status, binge drinking, and exercise were first transformed to dummy-like variables, with the new score ranging from 0 to 1. In each case, the least health-promoting alternative was recoded as 0 and the most health promoting alternative as 1. The values of the intermediate responses were interpolated, maintaining equal intervals between responses. For example, in the case of healthy diet for which there were five response alternatives, the least desirable alternative, “not at all,” was coded as 0, the most desirable alternative, “completely,” was coded as 1, while the middle response, “moderately,” was coded as 0.5. These scores were then summed to create the aggregated index, the score of which ranged from 0 to 5. Higher scores on this index indicated more favorable health behaviors.

## Results

**Figure 1:**
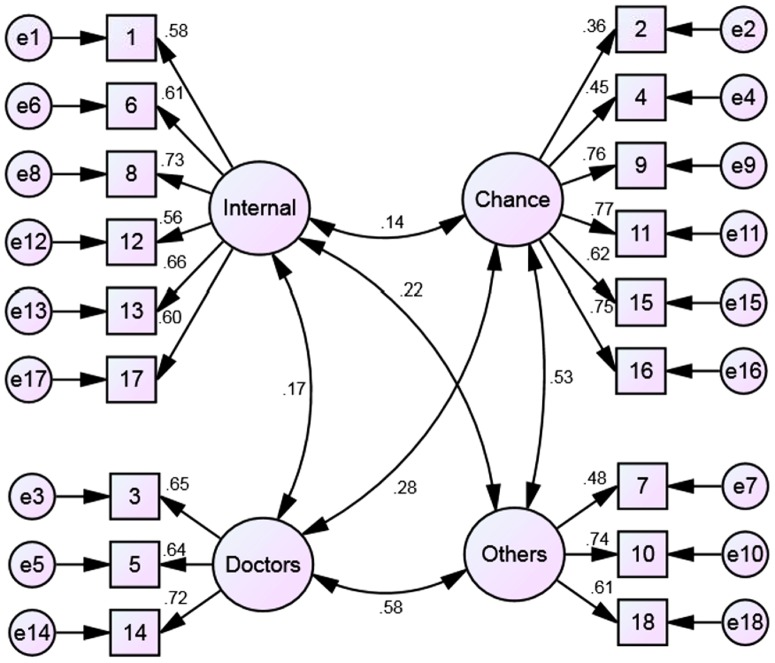
Structure of the MHLC Form C as hypothesized by the original test developers (displayed numbers are standardized regression weights in the present sample).

First, a four-factor structure with covariances among all factors (Figure 1) was tested using confirmatory factor analysis. The data showed inadequate fit for this model (χ^2^ = 397.3; p<.001; p _Bollen–Stine bootstrap_ = .002; CMIN/DF = 3.1; CFI = .86; RMSEA = .08; PCLOSE<.001; GFI = .89; TLI = .83) thus indicating that the original factor structure hypothesized by the test developers could not have been reproduced in this particular sample.

To discover what other factor structure would be more appropriate for this sample, a principal component analysis was conducted. The Bartlett's test of sphericity was significant (p<0.001) and the Kaiser–Mayer–Olkin measure of sampling adequacy for the MHLC-C was 0.77 indicating acceptable sampling. Results of the parallel analysis supported the extraction of three components as the eigenvalues of the fourth and fifth components from the first principal component analysis (with eigenvalues over 1.0) remained below the values produced by the parallel analysis (1.12 vs. 1.25 and 1.01 vs. 1.20, respectively). The second principal component analysis extracting only three components, accounted for 48.6% of the variance. Items of the Chance subscale loaded on Component 1 (loadings between .53 and .80), those of the Internal subscale on Component 2 (loadings between .66 and .77), while the items of the Doctors and Others subscales on Component 3 (loadings between .45 and .75). Loadings, eigenvalues, and explained variances for the exploratory analysis are displayed in Table 1. We can conclude that the solution emerged in the present study is very similar to that of the original test developers with the exception that the items of the two shorter subscales loaded onto one common component (from now on Doctors and Others subscale).

**Table 1:** Factor loadings from the second principal component analysis (Oblimin rotation with fixed number of factors).

Items (with their original subscale)	I.	II.	III.
Item 11 (Chance)	**.81**	.11	.24
Item 16 (Chance)	**.74**	.09	.32
Item 9 (Chance)	**.73**	.16	.33
Item 15 (Chance)	**.71**	−.10	.14
Item 4 (Chance)	**.59**	.04	.10
Item 2 (Chance)	**.53**	.02	−.05
Item 8 (Internal)	.16	**.77**	.11
Item 13 (Internal)	.19	**.73**	−.05
Item 6 (Internal)	−.06	**.69**	.17
Item 1 (Internal)	−.11	**.68**	.19
Item 17 (Internal)	.16	**.67**	.11
Item 12 (Internal)	−.12	**.66**	.22
Item 14 (Doctors)	.04	.10	**.75**
Item 3 (Doctors)	.10	.14	**.71**
Item 5 (Doctors)	.06	.08	**.70**
Item 10 (Others)	.35	.11	**.64**
Item 18 (Others)	.37	.19	**.51**
Item 7 (Others)	.26	.19	**.45**
Eigenvalues from the present data set	4.03	2.82	1.90
Random data eigenvalues from the parallel analysis	1.49	1.37	1.32
Explained variance	22.4%	15.6%	10.6%

Note. The dominant factor loading for each item is highlighted with bold fonts.

Internal consistency of all three subscales was appropriate. More detailed results of the item analysis and the intercorrelations among the subscales are presented in Table 2. Descriptive results of the four subscales for the total and the three subsamples can also be found in Table 2. Comparison of the subsamples showed that there was no significant difference between the cancer and cardiovascular patient groups concerning the Chance subscale (Mann-Whitney U = 6829.0; p = .495; r = .04). In the contrary, significant differences were observed with regard to the Internal (Mann-Whitney U = 6119.5; p = .045; r = .10) and the Doctors and Others (Mann-Whitney U = 5006.5; p<.001; r = .25) subscales; in both cases, patients with cardiovascular disorders reached higher scores.

**Table 2:** Internal consistency, descriptive statistics, and intercorrelations for the subscales of the Hungarian version of the MHLC Form C.

	Number of items / alpha	Corrected item-total correlations	M (SD)				Internality	Chance
			Total sample (N = 374)	Patients with cancer (n = 125)	Patients with CVD (n = 121)	Patients with other diseases (n = 128)		
Internality	6/.79	.52–.63	24.25 (6.23)	23.34 (6.05)	24.67 (6.34)	24.73 (6.25)	-	-
Chance	6/.78	.36–.66	16.65 (6.94)	17.19 (7.06)	16.35 (6.75)	16.39 (7.02)	.05^NS^	-
Doctors and Others	6/.71	.35–.51	24.81 (5.65)	24.61 (5.81)	27.38 (4.34)	22.67 (5.64)	.21***	.29***

CVD: cardiovascular diseases;^ NS^non-significant; ***p<.001.

Concerning the relationships with the further scales and questions, the Internal subscale of the MHLC-C associated negatively with anxiety, depression, and illness intrusiveness, while it associated positively with the Internal score of Levenson's Locus of Control Scale, self-efficacy, and self-rated health. The Chance subscale of the MHLC-C related positively to the Chance and Others score of Levenson's Locus of Control Scale, and further related positively to depression and anxiety. Although very weakly, it also associated negatively with more favorable health behaviors. Finally, the Doctors and Others subscale of the MHLC Form C associated significantly and positively with the Internal, Chance, and Others scores of Levenson's Locus of Control Scale, self-efficacy, illness intrusiveness, and a stronger proneness to seek medical attention for health complaints. Strength and level of significance for each relationship as well as the comparison of the strength of each pair of relationships can be seen in Table 3.

**Table 3:** Relationship of the MHLC Form C subscales with the other variables.

	Form C of the MHLC Scales			Comparison of the coefficients‡
	Internality	Chance	Doctors and Others	
	(I)	(II)	(III)	
General locus of control / internal	.35***	.07^NS^	.30***	I>II, I = III, II<III
General locus of control / chance	.09^NS^	.65***	.23***	I<II, I<III, II>III
General locus of control / powerful others	.01^NS^	.43***	.26***	I<II, I<III, II<III
Anxiety	−.15**	.15**	.04^NS^	I<II, I<III, II = III
Depression	−.19**	.17**	.02^NS^	I<II, I<III, II<III
Self-efficacy	.21***	.04^NS^	.19***	I<II, I = III, II<III
Illness intrusiveness	−.19***	.06^NS^	.12*	I<II, I<III, II = III
Self-rated health	.18**	−.04^NS^	−.10†	I<II, I<III, II = III
Seeking medical attention	−.09^NS^	<.01^NS^	.26***	I = II, I<III, II<III
Health behavior index	.01^NS^	−.12*	.11†	I = II, I = III, II<III

NS non-significant;

† p<.1;

*p<.05;

**p<.01;

***p<.001;

‡ comparisons are based on Steiger's Z scores, equal sign means p>.05.

## Discussion

Throughout the past decades, a large body of research has investigated health-related control beliefs using some form of the Multidimensional Health Locus of Control Scales [1], [9]. Form C of the instrument was specifically designed to investigate health-related control beliefs of persons with an existing medical condition. Psychometric studies investigating this questionnaire showed ambiguous results concerning factor structure and reliability. Following the call of Luszczynska and Schwarzer [38], the aim of the present study was to contribute to a better understanding of possible cultural influences on the properties of the MHLC-C by providing preliminary data on the psychometric characteristics of an Eastern European adaptation.

Beyond the inevitable necessity of psychometric investigations in every culture prior to the examination of complex hypotheses regarding health and illness, the relevance of this article lies in the fact that it examines health locus of control in a culture where helplessness and external control beliefs, many years after the collapse of the communist dictatorship, are still quite general in most areas of life [39]—contrary to the societal context in which the original test was developed.

Our results show that, with a minor modification, the factor structure originally constructed can be applied to Hungarian society as well. At this point, we can only speculate why—similarly to the findings of Dahnke and his colleagues [40]—the items of the Doctors and the Others subscales did not differentiate from each other and composed a common factor. It seems to us reasonable to assume that the most relevant health-related assistance comes almost exclusively from health care professionals in the cultural context studied; therefore, the respondents were not able to distinguish between the efforts of physicians versus others. Further investigations should clarify whether the factor structure found in this sample is stable across samples from Hungary or the broader Eastern European region, or whether its deviation from the original factor structure can be traced back to some specific characteristics of the present sample.

Internal reliability of the three subscales was appropriate in the present sample; the alpha values were well above those found in the Chinese investigations [12], [13]. Our results concerning internal consistency are also in line with previous studies showing that the Internal and Chance subscales have adequate alpha values, while those of the shorter subscales are only around the .70 threshold [1], [10], [11]. Further, although the relationships of the MHLC-C subscales with the additional variables were usually weak, these patterns concerning the validity of the instrument are very similar to that of the original authors [1] and to previous findings from other Western countries [11], [41]. The results indicate that the Hungarian version of the instrument is substantially no less capable of operationalizing the health locus of control construct in the studied cultural context than in that of the original test development. These findings point in the direction that Form C of the MHLC Scales might be an appropriate assessment tool of health control beliefs outside the traditional Western societies as well.

Hence, it is possible that the reason for the clearly inadequate psychometric properties of the MHLC-C previously found in China is not to be found mainly in the learned helplessness construct or differences in general external control beliefs but around the individualism–collectivism polarity [38]. While Asian countries are typically collectivist cultures, Hungary is clearly an individualist society—in this regard at least—more similar to typical Western countries [42]. Another possibility is that the unsatisfactory reliability and ambiguous factor structure found in China should not be traced back to cultural differences but to target populations. Namely, both previously mentioned Chinese studies examined healthy pregnant women, which is a condition quite different from chronic illnesses such as cancer or diabetes usually studied when employing Form C of the MHLC Scales [43].

Finally, some limitations of the present study should also be noted. First, although the sample size in the present study was technically large enough to conduct the factor analyses, our sample was too small and heterogeneous to investigate the factor structure of the instrument separately for each different disease group. Given that the specialty of the investigated form of the Multidimensional Health Locus of Control Scales is that it assesses condition-specific health-related control beliefs, from the aspect of test adaptation, the present study should be seen as preliminary. Further studies with larger and more homogenous samples should investigate whether the factor structure emerged here also fits homogenous groups of patients suffering from different chronic illnesses.

In addition, since no standardized, brief questionnaire was available in Hungarian in the study period, the questions and the index score used for the evaluation of the respondents' health behaviors were ad hoc; therefore, the validity of these data is uncertain. Also, temporal stability of the instrument was not tested; further studies should compensate for this shortcoming of the present investigation. Lastly, although the present study was conducted in a post-communist country of Eastern Europe making it probable that its participants could be characterized by weaker general (non-health specific) internal control beliefs than their western counterparts, this assumption could not have been tested formally in the absence of a western study group or available, current descriptive data for the relevant scales (general control beliefs and self-efficacy) from the West. Thus, final conclusions cannot be drawn on the question as to what extent health-related locus of control is affected by general control beliefs of a certain society. Therefore, further studies from the region should provide an explicit examination of the differences in general control beliefs between Western and post-communist countries, as they also need to examine the generalizability of the present findings by testing the invariance of the emerged factor structure of the MHLC-C across different countries.

However, we believe that investigating the psychometric properties of the MHLC Scales before their employment in a new region is a vital part of the responsible use of this psychological assessment tool in medicine, and we hope that the first steps towards this direction made in this paper will facilitate the conduction of more robust and informative cross-cultural studies on the relationship of general and health-related control beliefs.

## Supporting Information

Text S1 **Full text of the Hungarian version of the Multidimensional Health Locus of Control Scale Form C.**
(DOC)
