# Supplementary material for: Psychometric Properties of the Multidimensional Health Locus of Control Scale Form C in a Non-Western Culture
Source: PLoS One. 2014 Sep 9;9(9):e107108. doi: 10.1371/journal.pone.0107108 (PMC4159290; doi:10.1371/journal.pone.0107108)
Supplement: Text S1 — Full text of the Hungarian version of the Multidimensional Health Locus of Control Scale Form C. (DOC) [file pone.0107108.s001.doc]

Többdimenziós Egészség-kontrollhely Skála – C-Forma (MHLC-C-H)[[1]](#footnote-2)

Az alábbiakban olvasható minden mondat egy, az Ön egészségi állapotával kapcsolatos állítást tartalmaz, melyekről el kell döntenie, hogy egyet ért-e velük vagy sem. Minden egyes állítás mellett talál egy skálát, ahol az „1” azt jelenti, hogy egyáltalán nem ért egyet, a „6” pedig hogy teljesen egyetért. Arra kérjük, hogy minden állítással kapcsolatban karikázza be azt a számot, amely leginkább tükrözi egyetértése mértékét! Minél inkább egyetért tehát az adott kijelentéssel, annál nagyobb számot karikázzon be; és minél kevésbé ért egyet egy állítással, annál kisebb értéket jelöljön meg! Kérjük, figyeljen arra, hogy MINDEN EGYES ÁLLÍTÁSNÁL válasszon, és hogy CSAK EGY számot karikázzon be! A teszt az Ön személyes véleményét méri, ezért természetesen nincsenek jó vagy rossz válaszok!

|  | Egyál-talán nem értek egyet | In-kább nem értek egyet | Vala-melyest nem értek egyet | Vala-melyest egyetértek | In-kább egyetértek | Telje-sen egyet értek |
| --- | --- | --- | --- | --- | --- | --- |
| 1. Ha az állapotom romlik, rajtam múlik, hogy milyen hamar érzem ismét jobban magam. | 1 | 2 | 3 | 4 | 5 | 6 |
| 2. Ami az állapotomat illeti: lesz, ami lesz. | 1 | 2 | 3 | 4 | 5 | 6 |
| 3. Ha rendszeresen látogatom az orvosomat, kevesebb problémám lesz az egészségi állapotommal. | 1 | 2 | 3 | 4 | 5 | 6 |
| 4. A legtöbb dolog, ami hatással van az állapotomra, véletlenszerűen történik. | 1 | 2 | 3 | 4 | 5 | 6 |
| 5. Bármikor rosszabbodik az állapotom, fel kell keresnem egy egészségügyi szakembert. | 1 | 2 | 3 | 4 | 5 | 6 |
| 6. Kifejezetten felelős vagyok az állapotom romlásáért vagy javulásáért. | 1 | 2 | 3 | 4 | 5 | 6 |
| 7. Más embereknek fontos szerepe van abban, hogy az állapotom javul, változatlan marad vagy rosszabbodik. | 1 | 2 | 3 | 4 | 5 | 6 |
| 8. Az én hibám, ha az állapotom rosszabbodik. | 1 | 2 | 3 | 4 | 5 | 6 |
| 9. A szerencsének nagy szerepe van abban, hogyan javul az állapotom. | 1 | 2 | 3 | 4 | 5 | 6 |
| 10. Az, hogy az állapotom javuljon, mások erőfeszítésein múlik. | 1 | 2 | 3 | 4 | 5 | 6 |
| 11. Bármilyen javulás következik be az állapotomban, az a jó szerencsétől függ. | 1 | 2 | 3 | 4 | 5 | 6 |
| 12. A legfontosabb dolog, ami hatással van az állapotomra, az az, hogy én magam mit teszek. | 1 | 2 | 3 | 4 | 5 | 6 |
| 13. Akár javul, akár romlik az állapotom, az az én érdemem, illetve hibám. | 1 | 2 | 3 | 4 | 5 | 6 |
| 14. Pontosan követni az orvos utasításait a legjobb módja annak, hogy ne romoljon az állapotom. | 1 | 2 | 3 | 4 | 5 | 6 |
| 15. Ha romlik az állapotom, azt a sors akarta így. | 1 | 2 | 3 | 4 | 5 | 6 |
| 16. Ha szerencsém van, az állapotom javulni fog. | 1 | 2 | 3 | 4 | 5 | 6 |
| 17. Ha romlik az állapotom, az amiatt van, mert nem törődtem magammal eleget. | 1 | 2 | 3 | 4 | 5 | 6 |
| 18. A másoktól kapott segítségtől függ, milyen gyorsan javul az állapotom. | 1 | 2 | 3 | 4 | 5 | 6 |

1. Fordították a Tűzmadár Alapítvány munkatársai. Kapcsolat: dr. Konkolÿ Thege Barna, konkoly.thege.barna@gmail.com [↑](#footnote-ref-2)
